# A Joint Council for After-War Service

**Published:** 1920-05-22

**Authors:** 


### THE RED CROSS AND THE WHITE.

### A Joint Council for After-War Service.

On the outbreak of war in 1914 there were two Corporations authorised to engage in relief work among the sick and wounded men of His Majesty's Forces, viz., the British Red Cross Society and the Order of St. John of Jerusalem. These two bodies worked independently, but quickly came to the conclusion that everything must be done to co-ordinate their activities and to avoid waste and overlapping, and that to this end it was expedient to "pool" their funds and their efforts. A Joint War Committee was formed, together with a Joint Finance Committee, and it is under these two Committees that the voluntary work of relieving the sufferings of the sick and wounded men of His Majesty's Forces has been carried out during the past six years.

During that period a vast number of workers, both men and women, throughout the whole Empire, gave unwearying and devoted service to the cause, and it was natural that, when peace came, there should be a general feeling that an organisation such as this, founded on the voluntary efforts of those whose object was the relief of suffering and distress, should not be disbanded or allowed to disappear.

Moreover, there is still the aftermath of war to be dealt with. The Joint War Committee has been engaged since the armistice in carrying on certain of its War Departments which deal with work that must continue for some time yet to come, such as: The care of the sick and wounded men of His Majesty's Forces, whether still on the active list or demobilised. Such care as may still be necessary for those who have been prisoners of war. Assistance to orthopædic clinics and curative posts for the treatment of pensioners. Home service ambulance organisation. Red Cross and St. John War and Peace Library.

There are also other branches of work which can usefully be undertaken in time of peace by the Order of St. John and the British Red Cross Society, such as: The care of those suffering from tuberculosis, having regard in the first place to sailors and soldiers, whether they have contracted the disease on active service or not. Assistance, financial and otherwise, to the voluntary civil hospitals, in view of the strain put upon these hospitals by the war. Work parties to provide the necessary garments, etc., for hospitals and health institutions. Child welfare work. Assistance where required in all branches of nursing, health and welfare work ancillary to the Ministry of Health.

The two Corporations were anxious that these various branches of work should be carried on, and felt that for this purpose a governing body should be set up in place of the Joint War Committee, which had been created only for work in time of war. They have, therefore, by a formal agreement, established a Joint Council, on which both bodies have equal representation. This Council possesses under the old Charter of the Order of St. John and the newly extended Charter of the British Red Cross Society, full power to act in all matters connected with "the improvement of health, the prevention of disease, and the mitigation of suffering throughout the world." (Extract from Article XXV. of the Covenant of the League of Nations.)

The Joint Council has now begun its work, and makes an earnest appeal to the public for a continuance of that great and munificent support which carried the Joint War Committee through the years of war. During those years the sick and wounded men of His Majesty's Forces learnt to look upon the Red Cross as an emblem, not only of help and comfort, but also of the devotion of the British people for the men who were fighting in Britain's cause. It is our earnest hope that those who, in peace time, are in suffering or distress, will learn in the same way to look to the White Cross of St. John and the Red Cross for comfort and relief, and will feel, when helped by the Order of St. John and the British Red Cross Society, that they are not receiving anything in the nature of charity but only the just and proper tribute from those who are enjoying health and strength to their fellow men and women who are less fortunate than themselves.

For some branches of the work mentioned above—those in the first category—the Joint War Committee will continue to provide the necessary funds, either wholly or in part, for some time to come, but for all the other work of the Joint Council money is needed at once.

The greater the support that we receive from the public the better the work that we can do, and the more promptly we receive that support the sooner we can start our mission of healing and comfort. That is why we put forward this appeal, confident in the strength of our cause and in the generosity of the public that has never failed us yet.

Donations and subscriptions should be sent to The Joint Council Finance Committee, 19 Berkeley Street, London, W. 1. Cheques should be crossed "Lloyds Bank, Ltd."

Alexandra,
President of the British Red Cross Society.

Arthur,
Grand Prior of the Order of St. John of Jerusalem in England.

May 15, 1920.

